# Using visual lateralization to model learning and memory in zebrafish larvae

**DOI:** 10.1038/srep08667

**Published:** 2015-03-02

**Authors:** Madelene Åberg Andersson, Fredrik Ek, Roger Olsson

**Affiliations:** 1 Chemical Biology & Therapeutics, Department of Experimental Medical Science, Lund University, Lund, Sweden; 2 Medicinal Chemistry, Department of Chemistry and Molecular Biology, University of Gothenburg, Gothenburg, Sweden

## Abstract

Impaired learning and memory are common symptoms of neurodegenerative and neuropsychiatric diseases. Present, there are several behavioural test employed to assess cognitive functions in animal models, including the frequently used novel object recognition (NOR) test. However, although atypical functional brain lateralization has been associated with neuropsychiatric conditions, spanning from schizophrenia to autism, few animal models are available to study this phenomenon in learning and memory deficits. Here we present a visual lateralization NOR model (VLNOR) in zebrafish larvae as an assay that combines brain lateralization and NOR. In zebrafish larvae, learning and memory are generally assessed by habituation, sensitization, or conditioning paradigms, which are all representatives of nondeclarative memory. The VLNOR is the first model for zebrafish larvae that studies a memory similar to the declarative memory described for mammals. We demonstrate that VLNOR can be used to study memory formation, storage, and recall of novel objects, both short and long term, in 10-day-old zebrafish. Furthermore we show that the VLNOR model can be used to study chemical modulation of memory formation and maintenance using dizocilpine (MK-801), a frequently used non-competitive antagonist of the NMDA receptor, used to test putative antipsychotics in animal models.

Cognitive impairment is a core feature of neurodegenerative and neuropsychiatric disorders^1^,^2^. Despite the prevalence and impact on society, cognitive impairment remains an untreatable condition^3^. Today, there are several behavioral tests available when modelling cognitive impairment, including the frequently used novel object recognition (NOR) test^1^,^4^. The NOR test is regarded to reflect some aspects of human declarative memory and the unconditioned nature of the test makes it similar in some ways to memory tests in humans^5^. However, few animal models make it possible to study mechanisms involved in learning and memory in relation to brain asymmetry. Atypical functional hemispheric lateralization has been noted in schizophrenia and autism^6^, two complex disorders hypothesized to be diametric opposites joined by a spectrum of less severe disorders and normal cognition^7^. More elaborate and efficient behaviour-based screening models taking into consideration several aspects of learning and memory including brain asymmetry would likely identify cellular mechanisms involved in learning and memory that could help to bridge the gap in cognitive deficit treatments.

The NOR test is a simple, relatively short method, without confounding effects of external motivation rewards or punishment^5^. However using mammalian animal models to study functional brain lateralization on a cellular level is difficult^6^. In addition, utilization of mammalian models for large-scale screenings to find new biological mechanisms have proven to be inefficient, impractical, expensive, and highly questionable from an ethical perspective^8^. To overcome these limitations, zebrafish (*Danio rerio*) larvae are an attractive alternative. Zebrafish are being widely used to study brain functions and disorders as a feasible alternative to mammalian models, and have emerged as a powerful vertebrate organism for genetic research and drug discovery^1^,^3^,^4^,^9^,^10^,^11^. The popularity and strength of the zebrafish larvae model comes from the many valuable attributes, including reduced cost and time for husbandry and testing. Furthermore, transparency during larval stages allows microscopic viewing of non-invasive in-vivo studies^12^, making it possible to follow cellular communication in living animals. However, there are currently no cognitive tests available for zebrafish larvae that are comparable to the NOR model used in rodents. In a relatively short period of time a number of learning categories have been characterized in zebrafish larvae including habituation, dishabituation, sensitization, and classic conditioning^13^. All these learning paradigms address nondeclarative (implicit) memory as described for humans and rodents^14^,^15^. Nondeclarative memory refers to events (such as habits, priming, simple classical conditioning and non-associative learning) where experience alters the behavioral response non-consciously without providing access to any memory content. In contrast, a declarative (explicit) memory, as described for humans and rodents, refers to conscious recall, recollection, and associated feelings of familiarity^14^,^15^, such as novel object recollection^5^. At present there are no available assays used for zebrafish larvae that address declarative memory, such as the NOR model used in rodents. In addition, the traditional NOR model used in adult zebrafish is confounded by behaviors commonly used for the assessment of boldness and anxiety^4^. These behavioral expressions, including thigmotaxis (hugging the edges of a test chamber), freezing, hyperactivity, erratic movement, and accelerated movements^4^,^16^, are all characteristics used when describing individual stress coping^17^,^18^, and it could therefore be difficult to assign these behaviors to altered memory deficits as opposed to stress, boldness and anxiety when using the traditional NOR in adult zebrafish^1^.

It has long been recognized that the left and right hemispheres of the human brain differs both anatomically and functionally^19^. As recently as the 1970s the general consensus was that hemispheric specialization was a uniquely human trait^20^, however, today it is acknowledged that brain lateralization is a widespread and well-conserved phenomenon observed in several vertebrate and invertebrate species^19^,^20^,^21^,^22^,^23^,^24^. Apart from the evidence demonstrating the presence of brain lateralization across species, documentation also demonstrates fascinating similarities in brain function asymmetries between vertebrate species^19^. One particularly intriguing example is the visual specialization of the right hemisphere for face recognition in humans^25^. Similarly, a bias toward the left visual field in visual recollection of familiar and unfamiliar conspecifics is present in split-brained monkeys^26^, sheep^25^,^27^, toads, birds, amphibians^20^,^21^,^22^,^28^, and teleost fish^29^,^30^,^31^. Teleost species, including zebrafish, show assymetries in the use of the left and right eye systems when viewing conspecifics, where the left eye system (LES) is used when assessing novelty^1^,^8^,^31^. Furthermore, a study using lateral mutent zebrafish (with altered brain assyemtry) demonstrated that wild type larvae used the LES system to initially view their own reflection, and in mutents with altered brain assymetries the RES system was used to initially view its own mirror reflection^32^. On the other hand Miklósi et al.^33^ demonstrated contrasting results in adult zebrafish, where the LES system was used to view familiar conspecifics. Overall, these observations offers the opportunity to develop a unique NOR test based on visual lateralization in zebrafish larvae.

In the present work, we demonstrate a learning and memory paradigm that combines visual lateralization and the frequently used NOR test, the visual lateralization novel object recognition test (VLNOR), using the zebrafish larvae model. We generally use 10 dpf (days post fertilization) zebrafish larvae for behavioral pharmacology studies, including the VLNOR, because at 10 dpf the zebrafish larvae have a fully developed blood brain barrier^34^,^35^,^36^. Using the VLNOR test we demonstrate that zebrafish larvae have the capacity to obtain and recall memory of a novel object including both short- and long-term memory.

## Results and discussion

The VLNOR model takes advantage of the evolutionary well preserved visual bias of the LES when assessing novelty. After 30 min of acclimatization, novel objects were added to the experimental arenas. Individuals familiarized with the novel object for 8 min or 2 hours (for short- or long-term memory, respectively) after which the object was removed from the experimental arena. After a recovery period of 1, 2, 3 (short term), or 24 hours (long term) the novel objects were reintroduced in the wells. We used a camera to document visual bias using LES or the right eye system (RES) when observing the object. Data is presented as 2 min intervals from 0–2 min (T0), 2–4 min (T1), 4–6 min (T2) and 6–8 min (T3) after introduction of the novel object.

### Zebrafish larvae demonstrate short- term recollection of novel objects

**Figure 1:**
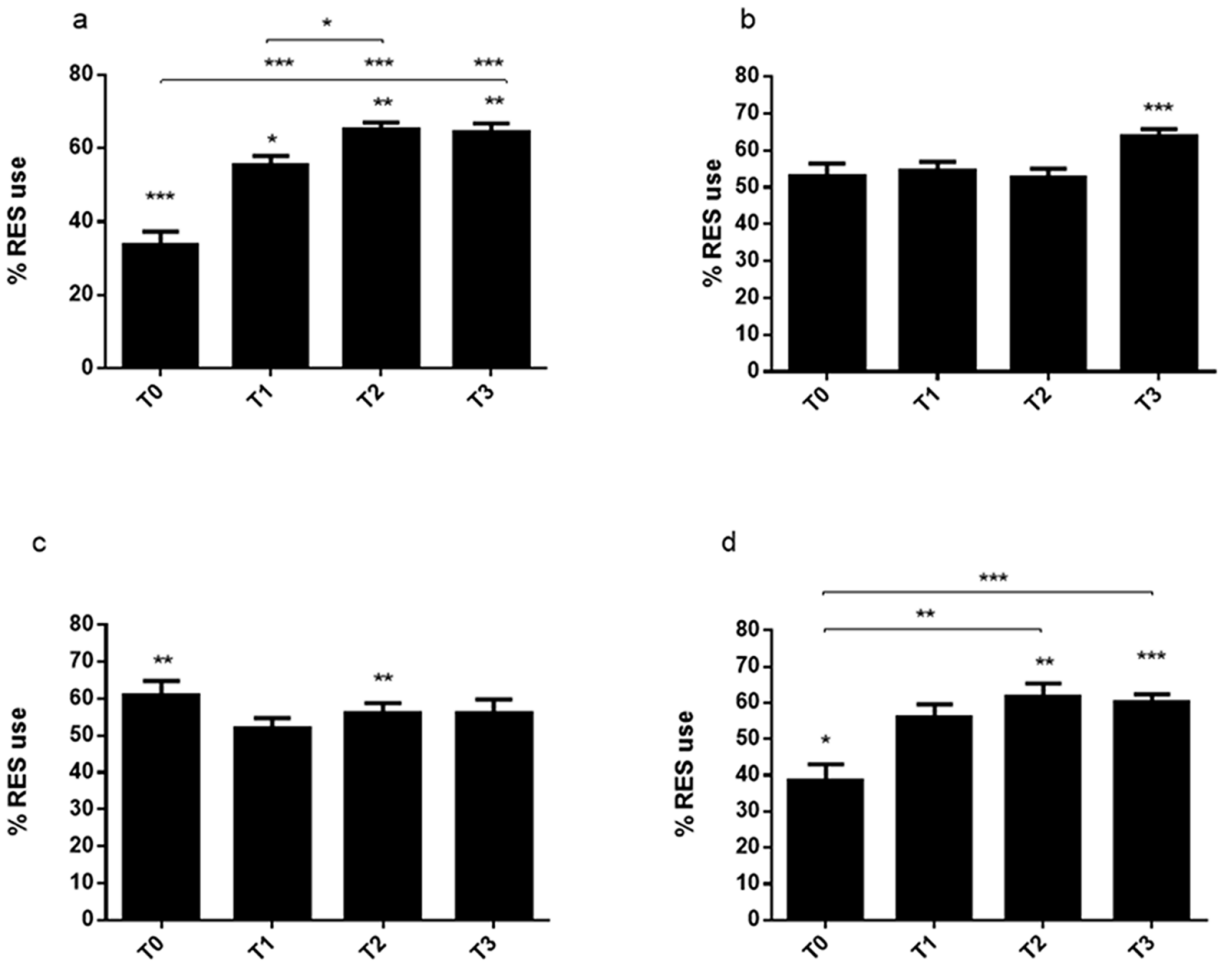
Memory formation and maintenance in 10 dpf zebrafish larvae, measured by visual lateralization in response to a novel object. Results are presented as mean values over 2 min intervals T0, 0–2 min; T1, 2–4 min; T2 4–6 min; T3, 6–8 min giving a total of 8 minutes per session. LES or RES preferences are marked by significance above the bars in the graphs. (a) RES use when presented with a novel object during the familiarization phase. (b) RES use when novel objects are reintroduced 1 hour after familiarization. Significant RES preference was detected at T3 (t = 6.78, p < 0.001). (c) Mean RES use when viewing a novel object when reintroduced 2 hours post familiarization. Significant RES preferences are observed at T0 (t = 3.09, p = 0.003) and T2 (t = 2.46, p = 0.018). (d) RES use when novel objects are reintroduced 3 hours after initial familiarization. A significant LES preference is detected at T0 (t = −2.52, p = 0.016) and RES preferences at T2 (t = 3.32, p = 0.002) and T3 (t = 4.46, p < 0.001). Significance *p < 0.05, **p < 0.001, ***p < 0.0001, computed using RMANOVA and Turkey's post-hoc test or a single sample t-test, tested against 50%. n = 48. Error bars indicate standard error of mean (SEM).

To assess the validity of the VLNOR model we first explored the capacity for zebrafish larvae to acquire, store, and recall information using visual lateralization as a read-out. Previous reports have demonstrated that teleost fish, including zebrafish, show differences in eye use when viewing novel environments or mirror reflections for the first time. For example, Sovrano and Andrew^31^ demonstrated that 8 dpf zebrafish preferentially used the LES during the first few minutes of viewing a reflection, and then changed to RES use, suggesting that the LES is used to assess novelty. Similarly, our results demonstrate that during the first encounter (familiarization phase) with a novel object the test individuals had a clear LES preference (calculated from a 50% threshold) during the initial 2 min (T0) of viewing (t-test, t = −4.58: p < 0.001). Furthermore, LES preference reduce over time (analysis of variance with repeated measures [RMANOVA)]: F_3.93_ = 34, p < 0.001) and use of the RES increased at T1 when the larvae displayed a RES preference versus a LES preference (t-test: T1, t = 2.22, p = 0.033). Furthermore, we found that the larvae had a statistically significant RES preference over the LES at T2 and T3 (t-test: T2, t = 7.73, p < 0.001; T3, t = 6.24, p < 0.001) when viewing the novel object. Thus the fish recognises the novel object within a period of 2-8 minutes (Figure 1A). These data indicate that zebrafish larvae display a visual bias toward the LES when initially viewing a novel object and that this bias is reduced presumably as the subjects establish whether the object has been seen before. Furthermore, it is interesting to note the distinct time frame within which individuals change eye system preference from the LES to the RES (i.e. when an object goes from being considered as unknown to familiar). To our knowledge this level of detailed novelty assessment in zebrafish larvae has not been previously described. However, in a study testing adult zebrafish in a one-trial memory test it was demonstrated that individuals had higher preference for exploration of novel objects during the first 5 min of the test, followed by a decrease and plateau of preference at 50% during the remainder of the test^37^. This suggests that the adult fish became familiar with the object within the initial 5 min which corroborate the data that we present here from tests in zebrafish larvae.

**Figure 2:**
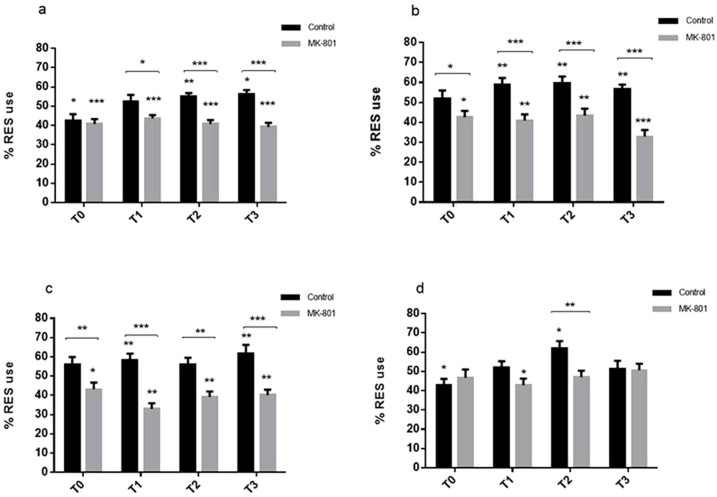
Pharmacologic modulation of NOR visualised by visual lateralization. 0.1 μM MK-801 is inserted into the wells 30 min prior to the familiarization phase. Results are presented as mean values over 2 min intervals: T0, 0–2 min; T1, 2–4 min; T2 4–6 min; T3, 6–8 min, (a total of 8 minutes per session). LES and RES preferences are indicated by significance above the graph bars. (a) RES use during the familiarization phase with novel objects after 30 min incubation in MK-801. Controls had significant LES preference at T0 (t = −2.29, p = 0.029) and RES preference at T2 (t = 2.74, p = 0.010) and T3 (t = 2.51, p = 0.017). MK-801-treated group had significant LES preference at T0, (t = −3.84, p = < 0.001) T1 (t = −3.71, p = < 0.001), T2 (t = −4.82, p < 0.001), and T3 (t = −5.11, p < 0.001). (b) RES use after reintroduction of novel objects (1 hour after familiarization). MK-801 has been present in the wells throughout the experiment. Controls display significant RES preference at T1 (t = 2.79, p = 0.008), T2 (t = 2.91, p = 0.007) and T3 (t = 2.41, p = 0.010) (c) RES during reintroduction of novel objects 2 hours after familiarization. Controls have significant RES preferences at T1 (t = 2.44, p = 0.020), and T3 (t = 2.59, p = 0.014). (d) RES use during novel object reintroduction 3 hours post familiarization. Controls display LES preference at T0 (t = −2.15, p = 0.039) and RES preference is observed at T2 (t = 3.26, p = 0.003). MK- 801 treated individuals display LES preference at T1 (t = −2.04, p = 0.015). Significance *p < 0.05, **p < 0.001, ***p < 0.0001,

Next, we were interested in the extent of the duration that the larvae were able to maintain the information obtained during familiarization. Individuals tested after 1 (Figure 1B) and 2 (Figure 1C) hours recovery did not display any significant LES use during novel object reintroduction. Instead there was a predisposition toward using the RES at all the given time points, suggesting that the test subjects could determine that the objects were familiar as they were presented into the wells. We further analysed the memory of the novel object in individuals that had had a 3 hour recovery period before reintroduction of the novel object. Collected data indicate that individuals no longer recognized the object, as demonstrated by the LES preference at T0 (Figure 1D) and considered the object to be novel when reintroduced. Similarly, as in the familiarization phase, RES use changed over time (RM ANOVA: F_3.93_ = 5.96, p = 0.007), where a higher use of the RES was detected towards the end of the test when the test subjects shifted to a clear RES preference after viewing the object.

Taken together these data demonstrate that zebrafish larvae consider the novel object to be novel during the first 2 min when viewing the object for the first time. Within 4 min they become familiarized with the object and are then able to retain information regarding the novel object for at least 2 hours. When the object was reintroduced after 3 hours the test subjects responded as if it was novel again.

### Pharmacological manipulation of glutamate neurotransmission reduces memory formation in zebrafish larvae

Intrigued by these findings we continued by investigating the possibility of using VLNOR to evaluate if a pharmacological modulation of the glutamatergic system would cause impairment of zebrafish larvae memory formation, by administrating the non-competitive *N*-methyl-*D*-aspartate (NMDA) receptor antagonist MK-801. Overwhelming evidence has indicated the role of NMDA receptors in processes of learning and memory^38^. The role of the NMDA receptors in learning and memory seems to be a well-conserved function throughout the animal kingdom^38^ including humans^39^, and administration of NMDA receptor antagonists has been demonstrated to impair learning performance in mammals and teleost fish^38^,^40^,^41^,^42^,^43^,^44^,^45^,^46^,^47^,^48^.

Addition of 0.1 μM of MK-801 to the well did not alter spontaneous behaviors in zebrafish larvae. Cognitive deficits created by blockage of the NMDA receptor were detected during the familiarization phase as individuals treated with MK-801 failed to familiarize with the object. Analysis of the RES demonstrated statistically significant decrease in the use of the RES in MK-801-treated individuals compared to control subjects (RMANOVA: F_1.64_ = 21.15, p < 0.001). An overall effect of time on the RES was detected (RMANOVA: F_3.192_ = 4.07, p = 0.007) where T1 (Turkey's post-hoc: p = 0.015), T2 (Turkey's post-hoc: p = 0.036) and T3 (Turkey's post-hoc: p = 0.046) values showed significantly higher RES use compared to T0 values. Furthermore, analysis demonstrated a statistically significant interaction effect of time and treatment (RMANOVA: F_3.192_ = 4.47, p = 0.004) on the RES where the control subjects demonstrated an increase in RES use over time; T1 (Turkey's post-hoc: p = 0.029), T2 (Turkey's post-hoc: p = 0.002) and T3 (Turkey's post-hoc: p < 0.001) values evidenced significantly higher RES use compared to T0 values (Figure 2A).

When testing short-term memory our results demonstrate that MK-801-exposed individuals neither formed nor maintained memory of the novel object in terms of LES preference at 1 hour recovery (t-test: T0, t = −2.39, p = 0.022; T1, t = −3.12, p = 0.004; T2, t = −2.14, p = 0.039, T3, t = −5.47, p < 0.001) and 2 hours recovery (t-test: T0, t = −2.33, p = 0.026; T1, t = −5.81, p < 0.001; T2, t = −3.41, p = 0.0012, T3, t = −3.52, p = 0.0013). Data from experiments performed with 1 (RMANOVA: F_1.59_ = 38.13, p < 0.001) and 2 (RMANOVA; F_1.59_ = 32.38, P < 0.001) hour recovery periods demonstrate that there were statistically significant effects of treatment, where controls had higher RES use compared to the MK-801-treated individuals (Figure 2B–C). Interestingly, with a predisposition towards LES preference, MK-801-treated individuals regarded the objects as novel throughout the sessions, suggesting a specific inhibition of learning rather than an effect of disorientation which would randomize LES/RES in a 50% ratio.

After 3 hours recovery, the controls demonstrated behavior similar to individuals that had encountered objects for the first time; thus these individuals did not recognize the objects. A statistically significant effect of treatment (RMANOVA: F_1.54_ = 6.74, p = 0.012) was detected where controls had higher RES use compared to the MK-801-treated group. An overall effect of time was observed (RMANOVA: F_3.162_ = 3.44, p = 0.003), where RES was higher at T2 compared to T0 (Turkey's post-hoc: p = 0.011) and T1 (Turkey's post-hoc: p < = 0.016). There was an effect of treatment on RES use but the MK-801-treated subjects did not increase RES usage over time, and the objects were seemingly considered as novel throughout the session. However, slightly higher RES use was detected during this time, indicating that the effect of the treatment could be wearing off (Figure 2D). Blockage of the NMDA receptor has been shown to cause deficits in memory formation in a number of different species^38^,^40^,^41^,^42^,^43^,^44^,^45^,^46^,^47^,^48^. NMDA receptor antagonists impaired adult zebrafish learning and memory in a passive avoidance test^41^, and were shown disrupt habituation in zebrafish larvae^10^. Using the VLNOR test, we demonstrated that MK-801-treated larvae viewed the objects as novel throughout the experiment. Similarly, as has been demonstrated in mice^45^,^49^, rats^47^ and teleost fish^40^,^42^, chemical modulation of the NMDA receptors blocked formation of memories in zebrafish larvae, as assessed using the VLNOR test.

### Effects of NMDA receptor antagonist MK-801 on memory retrieval

The confirmation that MK-801 altered locomotor performance in the VLNOR test, led us to investigate the effects of MK-801 on memory retrieval, a measure of particular importance when studying neurodegenerative diseases. Human research suggests that NMDA receptor antagonism may selectively impair memory formation of non-spatial information, but not the retrieval of the information already learned^39^,^50^.

**Figure 3:**
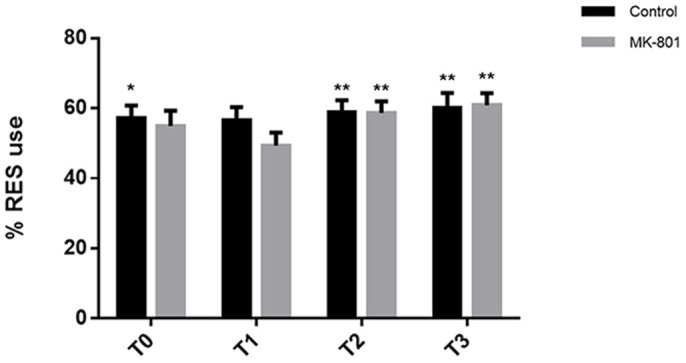
The effects of NMDA receptor antagonist MK-801 (0.1 μM) treatment on long term memory retrieval in 10 dpf zebrafish. Individuals were familiarized with novel objects for 2 hours in a massed paradigm. Individuals were moved to a fresh media without treatment for 23 hours, after which MK-801 was administered. Reintroduction of the objects occurred 1 hour after MK-801 administration. Controls have a significant RES preference at T0 (t = 2.00, p = 0.051), T2 (t = 3.44, p = 0.022) and T3 (t = 4.26, p = 0.023). MK-801-treated individuals displayed RES preference at T2 (t = 3.23, p = 0.012) and T3 (t = 3.47, p = 0.003). Significance *p < 0.05, **p < 0.001, ***p < 0.0001, computed using single sample t-test tested against 50%. For control and MK-801 treated groups, n = 36. Error bars indicate SEM.

We tested the effects of MK-801 on long-term memory retrieval. For this experimental setup, the individuals were allowed to familiarize with the object for 2 hours after which the objects were removed. MK-801 (0.1 µM) was added to the wells 23 hours after the familiarization phase, 1 hour before reintroduction of the objects into the wells (24 hours after familiarization). Treatment with MK-801 did not affect memory retrieval of the object. Both controls and MK-801-treated subjects recognized the objects as they were reintroduced 24 hours after familiarization (Figure 3). These results further confirm that the NMDA receptor antagonist MK-801 does not affect memory retrieval in short- or long-term memory assays.

We were also interested in testing the effects of MK-801 on short-term memory retrieval. Here we allowed both groups to become familiarized with the object prior to MK-801 administration. The familiarization phase was designed to exclude differences in memory formation prior to the addition of MK-801. There were no statistically significant differences between the groups at the baseline assessment for familiarization, demonstrating that subjects in both groups were able to normally form memories prior to any treatment. MK-801 (0.1 μM) was added directly after familiarization. The objects were then reintroduced at 1, 2, or 3 hours after familiarization.

There were no statistically significant differences between the controls and the MK-801-treated groups in any of the experiments conducted (1, 2, or 3 hour recovery tests), and all groups demonstrated the same behavior as presented by the controls (supplementary figure S1A–D). This suggests that, in zebrafish larvae, MK-801 does not influence retrieval of memories obtained prior to the treatment. Previous studies in humans and rodents have demonstrated that chemical modulation of the NMDA receptors using the NMDA receptor antagonist ketamine impairs formation of memories but does not affect the retrieval of established memories^39^,^48^,^50^,^51^. In contrast, few studies investigate the effect of MK-801 on memory retrieval using animal models, and in rodent research it has been demonstrated that, as opposed to ketamine, MK-801 treatment resulted in disrupted memory retrieval^52^,^53^. Furthermore, memory retrieval was impaired due to MK-801 in a T-maze test conducted in adult zebrafish when administered after familiarization or prior to memory retrieval^40^. Interestingly, using the VLNOR test we demonstrate that a blockage of the NMDA receptor using MK-801 does not disrupt memory retrieval in zebrafish larvae. To rule out the possibility that memory retrieval was not affected due to a low dose of MK-801, we repeated the memory retrieval experiment using a higher dose.

When using 1 μM of MK-801 the results were consistent with those attained following administration of the lower dosage (0.1µM); thus no impairment of memory retrieval was seen 1 hour post the familiarization phase (supplementary figure S2B). There were no statistically significant differences in RES use between the control and the MK-801-treated groups at either the familiarization phase (supplementary figure S2A) or 1 hour after familiarization (supplementary figure S2B). However, as has been previously described, a statistically significant main effect of time was observed during the familiarization phase (RMANOVA: F_3.116_ = 6.82, p < 0.001) where RES use increased over time.

### Zebrafish larvae demonstrate long-term novel object recollection

Unlike short-term memory, long-term memory formation has been shown to be protein synthesis dependant in a number of different organisms including teleost fish^10^,^54^,^55^,^56^. Literature reporting on zebrafish larvae has demonstrated that in cognitive tasks such as habituation^10^ and associative learning^54^, larvae are competent in long-term memory recollection. Furthermore it was also shown in these reports that the long-term memory was associated with protein synthesis. To determine if the VLNOR test could be used to study long-term memory, and at the same time determine if novel object recollection in zebrafish larvae requires protein synthesis, we used cycloheximide (CHX) (10 μM), to block protein synthesis. The VLNOR experimental procedure was slightly changed and individuals were trained with the novel object for 2 hours. CHX was added to the wells prior to familiarization with the objects and was present during the familiarization phase. After the familiarization phase the objects were removed from the wells and the media in the wells were changed and replaced with new E3 media. Individuals were tested again for novel object recollection 24 hours after familiarization.

**Figure 4:**
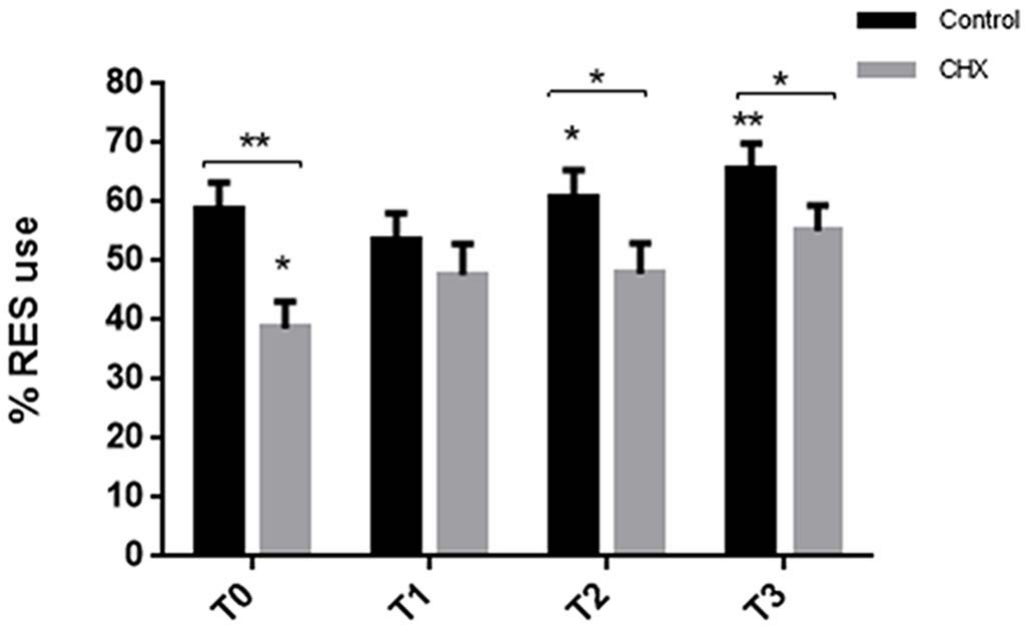
10 dpf zebrafish larvae demonstrate protein synthesis-dependent long-term NOR. Controls and CHX treated (10 μM) individuals were familiarized with novel objects for 2 hours in a massed paradigm. Individuals were moved to a fresh media without treatment for 24 hours and then reintroduced with the objects. CHX-treated individuals show LES preference at T0 (t = −2.49, p = 0.018). Controls had RES preferences at T2 (t = 2.46, p = 0.019) and T3 (t = 3.74, p < 0.001). Significance *p < 0.05, **p < 0.001, ***p < 0.0001, computed using a single sample t-test tested against 50%. For control and CHX treated groups, n = 36. Error bars indicate SEM.

As described in Figure 4, the zebrafish larvae had the capacity to retain information that they had obtained during the 2 hour familiarization phase 24 hours earlier. In accordance with the general consensus in the literature^10^,^54^,^55^,^56^, we demonstrate that protein synthesis is essential for long-term memory formation of novel objects in zebrafish larvae. There was a statistically significant difference between treatments (RMANOVA: F_1.53_ = 8.99, p = 0.004) on RES use, where CHX-treated individuals behaved as if though the object was novel (Figure 4). To test if differences in long-term memory were an effect of protein syntheses rather than a behavioral effect of the substance, we tested the effects of CHX on short-term memory. CHX was added to the wells prior to familiarization and the novel objects were added for the duration of 8 min. The objects were removed and individuals were given 1 hour of recovery before the objects were reintroduced into the system. Blockage of protein synthesis did not disrupt memory formation or short-term memory retrieval in zebrafish larvae (supplementary figure S3A–B). While it is clear that long-term memory requires new protein synthesis, far less is known regarding the identity of the proteins involved in memory maintenances^54^.

Furthermore, interestingly, CHX-treated larvae demonstrated a learning curve during the familiarization phase, during the short term memory test, that was consistent to that demonstrated by the controls. However in the long term memory test the learning curve for the CHX-treated individuals (that did not hold recollection of the object 24 hours post familiarization) is not as pronounced. The longer exposure time with CHX (during long term memory experiments) seems to impair learning performance at later occasions post treatment, an effect not seen in the short term paradigm. This is an interesting finding and further tests to analyse the effects of protein synthesis inhibition on learning are required.

## General conclusions

The VLNOR test takes advantage of an evolutionary conserved phenomenon, visual lateralization, to study memory formation, maintenance and retrieval in zebrafish larvae. The VLNOR test is not confounded by behaviors characteristic of the assessment of boldness or anxiety which makes it a good model to study learning and memory. In addition, the VLNOR test makes it possible to study learning and memory mechanisms different to those featured in the currently available rodent (e.g., functional lateralization) and zebrafish larvae (e.g., habituation, sensitization, and conditioning paradigms) models. Our results demonstrate that zebrafish larva have a predisposition to LES preference when initially encountering an object. Individuals change to a RES preference during the session, presumably when they determine that the object is familiar. We demonstrate that 10 dpf zebrafish larvae possess the capacity for short- and long-term memory of objects, and that long-term memory of the novel object is protein synthesis dependent. Furthermore, we show that the VLNOR test can be used to study chemical modulation of memory formation and maintenance using MK-801, a frequently used chemical to generate psychosis-relevant features in animal models to evaluate antipsychotics. Interestingly, we also demonstrate that the VLNOR test can be used to study memory retrieval, making this model highly attractive for the study of neurodegenerative diseases. We have focused the development of the VLNOR test for zebrafish larvae. Furthermore, considering that visual lateralization has been observed in several vertebrate and invertebrate species, and that brain hemisphere lateralization has been associated to both autism and schizophrenia, the VLNOR holds the potential for a number of different applications.

## Experimental procedures

### Animal ethics

This study was carried out in strict accordance with the national legislation of Sweden. All procedures were approved by the ethical committee in Malmö-Lund (permit, M116-12).

### Fish maintenance

The zebrafish larvae used in this study were from inter-crosses of the wild type AB strain. Embryos were collected and raised in a 14:10 hour light/dark cycle at 28°C on Petri dishes containing E3 media until they were 5 dpf. At the age of 5 dpf the larvae were transferred into 0.8 L aquaria and placed in an Aquaneering, Inc. (San Diego, CA) recirculating system held at 26 ± 1.5°C where feeding was initiated. Larvae were fed with a commercial larval diet, ZM000 (ZM Fish Food & Equipment, Winchester, UK), four times daily until the age of 10 dpf. Behavioral experiments were conducted at 10 dpf.

### Pharmacology

For pharmacological experiments MK-801 (M-107, Sigma-Aldrich, St. Louis, MO), and CHX (C7698, Sigma Aldrich) were used. Compounds were dissolved in 100% dimethyl sulfoxide and administrated in the final concentration of 0.1 μM or 1 μM MK-801 and 10 μM CHX via the E3 medium. When studying the effects of MK-801 on learning the MK-801 was administrated to the wells 30 min prior to the familiarization session. For the memory retrieval study using MK-801, the substance was added to the wells either directly after familiarization (short-term memory) or 1 hour prior to the reintroduction of the novel objects (long-term memory). CHX was added 30 min prior to the familiarization session and for the individuals that were tested 24 hours later the CHX was removed directly after the familiarization phase.

### Experimental setup and video recordings

Larvae were trained and tested at the density of one per microtiter well (round wells) in 2 ml E3 medium. Two 12 well microtiter well plates were tested parallel in the behavioral chamber. The behavioral chamber consisted of a 30 fps digital camera (Genie HM640, Teledyne DALSA, Waterloo, Canada) connected to a computer set up with video recording software (CamExpert v7.00.00.0912, Teledyne DALSA, Waterloo, Canada; Labview™ 2011 v11.0, National Instruments, Austin, TX). To maintain the environment in the wells at 27°C the microtiter plates were placed parallel to each other in a water bath containing a temperature control unit (Neoheater 25 W thermostat, AQUAEL, Warsaw, Poland). The water bath with the test subjects was placed on a light box, containing LED strips (SMD5050 flexible infrared 850 nm tri-chip) which were used to illuminate the microtiter plates. The behavioral chamber was illuminated from above with five LED spotlights at 15 lux, and a mirror was positioned at a 45° angle above the light box, enabling video recording. For a schematic image of the experimental setup see supplementary figure S4.

All individuals were observed for abnormal swimming behavior and body deformities prior to initiation of the novel object assay. Damaged individuals were removed and replaced. All assays were performed in triplicate experiments.

### Visual lateralization novel object model

Larvae were trained and tested for novel object recollection at 10 dpf. In total 24 (12 × 2) larvae were transferred, using 3 ml plastic pipettes, to microtiter well plates containing 2 ml of E3 medium. After being placed in the behavioral chamber all individuals were left to acclimatize for 30 min. After the acclimatization period, novel objects (10–100 μl pipette tips, painted black with nail polish) were introduced in the centre of the wells for the duration of 8 min after which the objects were removed. Novel objects were reintroduced with the individuals at 1, 2, or 3 hours after familiarization; again the objects were present in the wells for 8 minutes. Treatment with MK-801 was administered either prior to acclimatization or directly after familiarization with the novel object.

### Long term novel object recollection

The experimental setup to analyze the long term novel object capacity, of zebrafish larvae, was similar to that previously described in this paper. The microtiter plates were placed in the behavioral chamber and CHX was administrated. Individuals were left to acclimatize for 30 min after which the novel objects were introduced in the wells. Individuals were trained with the objects for 2 hours after which the objects were removed from the wells. After familiarization the individuals were transferred to wells containing 4 ml fresh E3 media (no CHX was added to the wells) and placed in an incubator for 24 hours. Temperature was held at 28°C. After 23.5 hours, all individuals were transferred to wells containing 2 ml fresh E3 media and then placed in the behavioral chamber. Individuals were left to acclimatize for 30 minutes, after which the novel objects were reintroduced for the duration of 8 minutes.

### Behavioral analysis

Cognitive behavioral analysis was conducted by viewing video recordings. Each exposure to the novel object consisted of 8 min that were divided into four blocks of 2 min each (T0, T1, T2, and T3). LES and RES postures were counted every 5 sec within the blocks, giving 24 left or right eye viewings for each block. Individuals that were not moving were not given a LES or RES score, and if there were not movement within the first 4 minutes the individual were removed from further analysis. In average, 2 individuals were immobilized and removed from each of the experiments. No more than 3 individuals were removed from any of the individual experiment; furthermore, when viewing these larvae it was noticed that the shape of the fish was bent indicating that injuries during handling could have caused the lack of movement in these fish. Postures (supplementary figure S5) of the larvae in relation to the novel objects had been explored in pilot experiments, and scoring of the LES and RES use was conducted according to these postures. An RES use index was calculated according to the formula described by^31^:
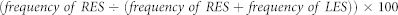
Values significantly higher than 50% would then indicate a RES preference and values significantly lower than 50% would indicate a LES preference.

### Statistics

Statistical analyses were performed using Statistica v12 (StatSoft, Tulsa, OK). Significant departures from 50% to determine left and right eye preferences were estimated by single-sample t-tests. Furthermore, data was analysed using RMANOVA where treatment and experimental replica (each experiment was performed in triplicates) were used as categorical predictors. Turkey's post hoc tests were used for further analysis of statistical differences.

## Author Contributions

M.Å.A., F.E. and R.O. took part in designing the project. M.Å.A. performed experiments, conducted statistical analysis, and prepared graphs and images. M.Å.A. and R.O. wrote the manuscript. All authors reviewed the manuscript.

## Supplementary Material

Supplementary Information Supplementary information
