# Supplementary material for: Using visual lateralization to model learning and memory in zebrafish larvae
Source: Sci Rep. 2015 Mar 2;5:8667. doi: 10.1038/srep08667 (PMC4345346; doi:10.1038/srep08667)
Supplement: Supplementary Information [file srep08667-s1.pdf]

Using visual lateralization to model learning and memory in zebrafish larvae

Authors:
Madelene Åberg Andersson
Fredrik Ek
Roger Olsson

Supporting information Figures and figure captions

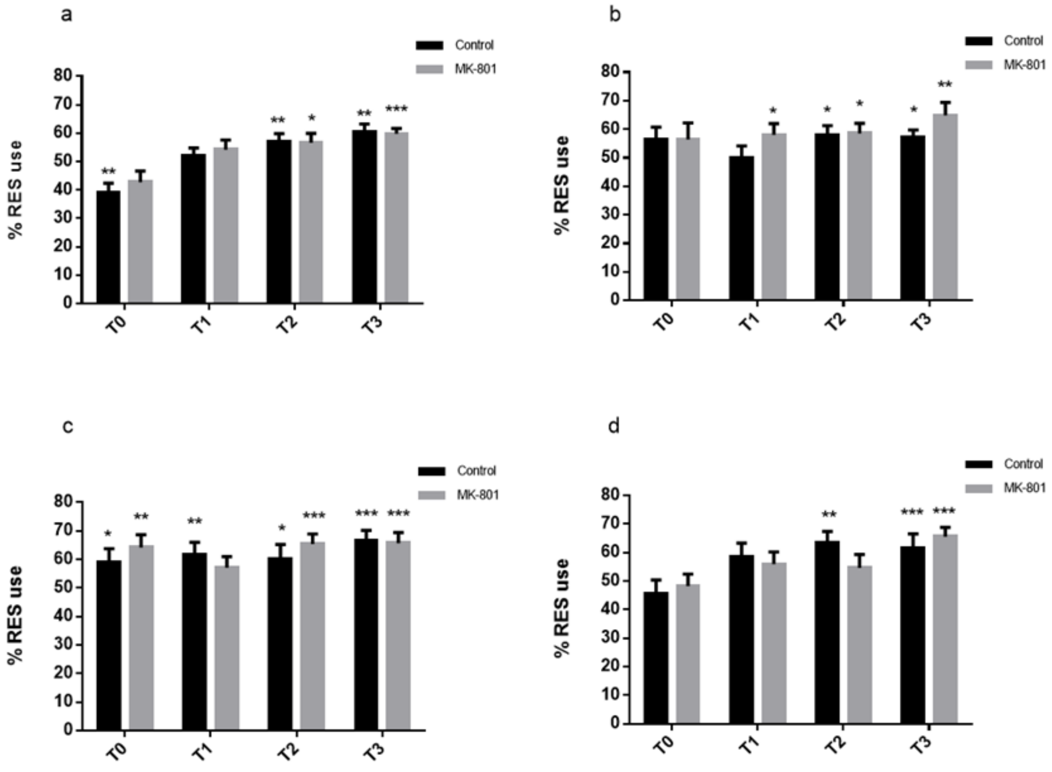

Figure S1. The effects of NMDA receptor antagonist MK-801 (0.1μM treatment after training) on RES use in 10 dpf zebrafish. MK-801 was administrated into the wells directly after removal of the novel objects from the familiarization phase. **a)** Amount of RES use during familiarization with the novel objects. LES preference was detected at T0 (control, t=-3.14, p=0.003; "MK-801", t=-1.64, p=0.063). RES preferences are detected at T2 (control, t=2.48, p=0.018; "MK-801", t=1.96, p=0.055) and T3 (control, t=3.69, p<0.001; "MK-801", t=5.05, p<0.001). **b)** Amount of RES use in controls and MK-801-treated individuals during reintroduction of the novel objects 1 hour after familiarization. Controls display RES preference at T2 (t=2.40, p=0.022) and T3 (t=2.70, p=0.011). MK-801 treated group displayed RES preference at T1 (t=2.07, P=0.047), T2 (t=2.45, p=0.020) and T3 (t=3.29, p=0.002). **c)** RES use in response to novel objects for controls and MK-801 treated individuals 2 hours after the familiarization phase. Significant RES preference was detected for controls at T1 (t=2.75, p=0.009), T2 (t=1.97, p=0.055) and T3 (t=4.49, p<0.001). Similarly, significant RES preference was demonstrated for the MK-801 treated group at T0 (t=3.18, p=0.003), T2 (t=4.33, p<0.001) and T3 (t=4.15, p<0.001). **d)** Measurements of RES use in control and MK-801 treated subjects during NOR 3 hours after familiarization. Controls had significant RES preference at T2 (t=3.34, p=0.002) and at T3 (t=2.36, P=0.025). MK-801-treated individuals displayed RES preference at T3 (t=4.69, p<0.001). Significance *p<0.05, **p<0.001, ***p<0.0001, computed using single sample t-test tested against 50%. For control and MK-801 treated groups, n=36. Error bars indicate SEM.

Using visual lateralization to model learning and memory in zebrafish larvae

Authors:
Madelene Åberg Andersson
Fredrik Ek
Roger Olsson

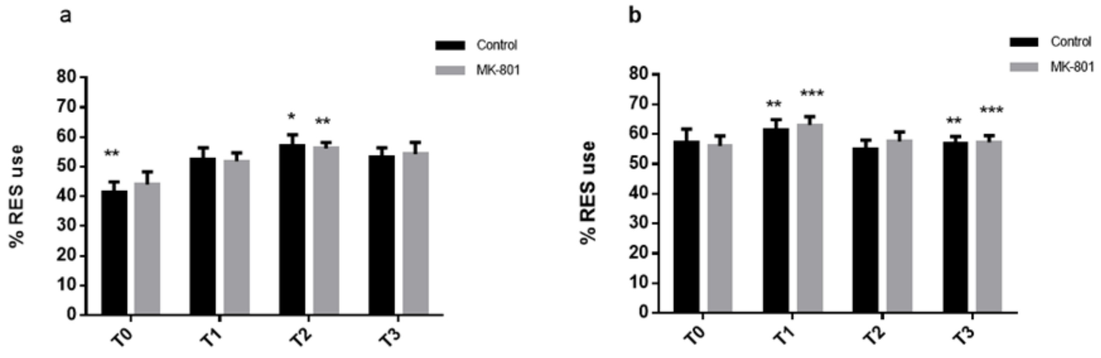

Figure S2. The effects of NMDA receptor antagonist MK-801 (1μM treatment after familiarization) on RES use in 10 dpf zebrafish. MK-801 was administrated into the wells directly after removal of the novel objects from the training session. **A)** Amount of RES use during training with the novel objects. Groups are denoted as control and MK-801 although no treatment had been added to the wells at this time. Controls displayed a significant LES preference at T0 (t=-2.46, p=0.019), and at T2 a significant RES preference was found (t=2.00, p=0.051). MK-801 treated individuals displayed significant RES use at T3 (t=3.28, p=0.002). **B)** Amount of RES use in controls and MK-801 treated individuals during reintroduction of the novel objects 1 hour after the familiarization phase. Controls display RES preference at T1 (t=3.02, p=0.003) and at T3 (t=3.06, p=0.004). MK-801 treated group displayed RES preference at T1 (t=4.94, p<0.001), and T3 (t=4.22, p<0.001). Significance *p<0.05, **p<0.001, ***p<0.0001, computed using single sample t-test tested against 50%. For control and MK-801-treated groups, n=36. Error bars indicate SEM.

Using visual lateralization to model learning and memory in zebrafish larvae

Authors:
Madelene Åberg Andersson
Fredrik Ek
Roger Olsson

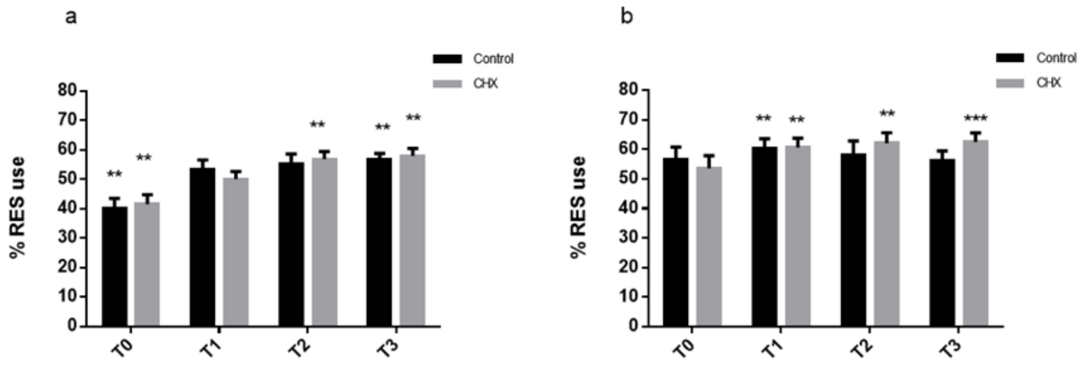

Figure S3. The effects of disrupted protein synthesis on memory formation and short-term memory maintenance in 10 dpf zebrafish larvae. CHX was added to the wells 30 min prior to the familiarization phase. **A)** RES use in controls and CHX-treated individuals during an 8 min familiarization phase. Significant LES preferences were found at T0 for both groups (control: t=-2.87, p=0.007; CHX: t=-2.66, p=0.011) and RES preferences at T2 (CHX: t=2.66, p=0.011), and T3 (control: t=2.87, p=0.007; CHX: t=3.14, p=0.003). **B)** RES use in controls and CHX treated individuals in response to NOR 1 hour after familiarization. Significant RES preference was detected for the controls at T1 (t=3.01, p=0.005). CHX-treated individuals display significant RES at T1 (t=3.29, p=0.002), T2 (t=3.66, p=0.001), and T3 (t=3.99, p<0.001). Significance *p<0.05, **p<0.001, ***p<0.0001, computed using a single sample t-test tested against 50%. For control and CHX treated groups, n= 36. Error bars indicate SEM.

Using visual lateralization to model learning and memory in zebrafish larvae

Authors:
Madelene Åberg Andersson
Fredrik Ek
Roger Olsson

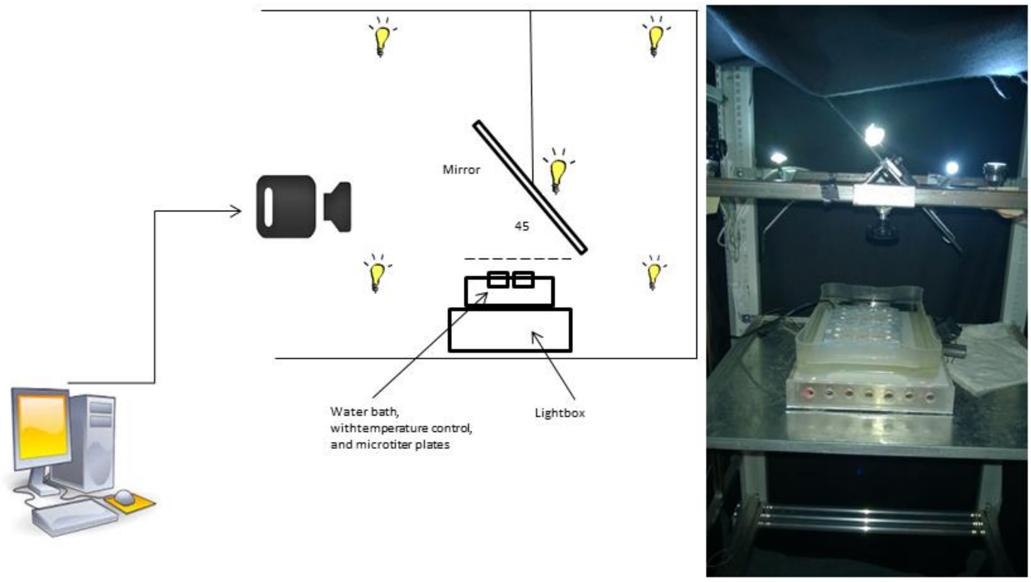

Figure S4. Schematic view of the testing apparatus for the behavioral analysis. This image was constructed by the first author of this manuscript, MÅA.

Using visual lateralization to model learning and memory in zebrafish larvae

Authors:
Madelene Åberg Andersson
Fredrik Ek
Roger Olsson

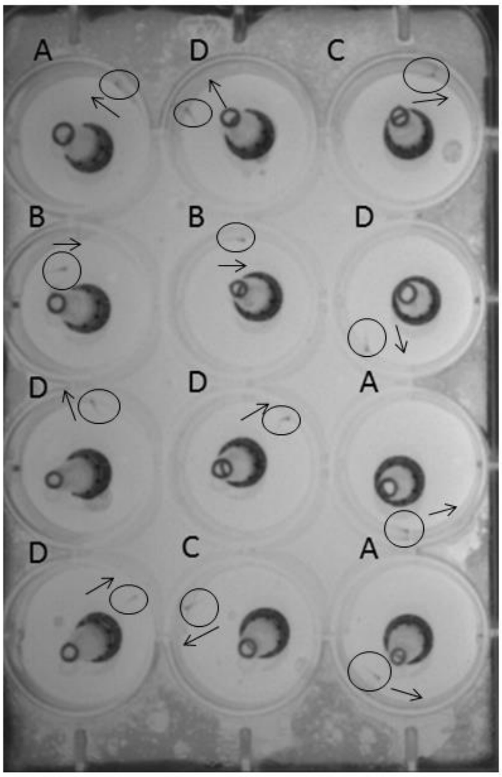

Figure S5. Digital image of the VLNOR, demonstrating zebrafish larvae postures in relation to the novel object. A) Parallel positioning to the object; LES view. B) Parallel positioning; RES view. C) Angled positioning; LES or RES view. D) Facing away from object; no score.
